# Protein Kinase D1 (PKD1) Is a New Functional Non-Genomic Target of Bisphenol A in Breast Cancer Cells

**DOI:** 10.3389/fphar.2019.01683

**Published:** 2020-01-31

**Authors:** Messaouda Merzoug-Larabi, Ilige Youssef, Ai Thu Bui, Christine Legay, Sophia Loiodice, Sophie Lognon, Sylvie Babajko, Jean-Marc Ricort

**Affiliations:** ^1^ Centre National de la Recherche Scientifique, CNRS UMR_8113, Laboratoire de Biologie et Pharmacologie Appliquée, Cachan, France; ^2^ École Normale Supérieure Paris-Saclay, Université Paris-Saclay, Cachan, France; ^3^ Centre de Recherche des Cordeliers, INSERM, Sorbonne Université, Université de Paris, Laboratoire de Physiopathologie Orale Moléculaire, Paris, France

**Keywords:** endocrine disruptor, bisphenol A, protein kinase D1 (PKD1), breast cancer, pro-tumorigenic factor, non-genomic target

## Abstract

Exposure to bisphenol A (BPA), one of the most widespread endocrine disruptors present in our environment, has been associated with the recent increased prevalence and severity of several diseases such as diabetes, obesity, autism, reproductive and neurological defects, oral diseases, and cancers such as breast tumors. BPA is suspected to act through genomic and non-genomic pathways. However, its precise molecular mechanisms are still largely unknown. Our goal was to identify and characterize a new molecular target of BPA in breast cancer cells in order to better understand how this compound may affect breast tumor growth and development. By using *in vitro* (MCF-7, T47D, Hs578t, and MDA-MB231 cell lines) and *in vivo* models, we demonstrated that PKD1 is a functional non-genomic target of BPA. PKD1 specifically mediates BPA-induced cell proliferation, clonogenicity, and anchorage-independent growth of breast tumor cells. Additionally, low-doses of BPA (≤10^−^^8^ M) induced the phosphorylation of PKD1, a key signature of its activation state. Moreover, PKD1 overexpression increased the growth of BPA-exposed breast tumor xenografts *in vivo* in athymic female Swiss nude (*Foxn1^nu/nu^*) mice. These findings further our understanding of the molecular mechanisms of BPA. By defining PKD1 as a functional target of BPA in breast cancer cell proliferation and tumor development, they provide new insights into the pathogenesis related to the exposure to BPA and other endocrine disruptors acting similarly.

## Introduction

According to the World Health Organization’s definition (2002), endocrine disruptors are chemical compounds that can interfere with the endocrine system and cause deleterious health effects to organisms or even their descendants. Among the most common endocrine disruptors, bisphenol A (BPA), used in epoxy resins and polycarbonate plastics, has been detected in biological fluids of most of the population worldwide, essentially due to oral contamination by ingestion of BPA-containing food and drinks (Pirard et al., 2012; Galloway et al., 2018). Epidemiological and experimental studies show that human BPA serum concentrations generally vary from 0.2 to 1.6 ng/mL^−1^ (0.88 to 7 nM), but may reach higher values in workers who manipulate high amounts of BPA (thermal paper or plastics industries) (Hines et al., 2017). Many adverse health effects, such as hyperactivity, obesity, fertility problems, enamel defects, and cardiac arrhythmia have been associated with exposure to BPA, especially during the perinatal period of life (Jedeon et al., 2013) (reviewed in Giulivo et al., 2016). Moreover, exposure to BPA has been associated with the recent increased incidence of prostate and breast cancer (reviewed in Seachrist et al., 2016).

Gestational or perinatal exposure of rodents and monkeys to BPA alters mammary gland development, increasing the risk of later onset of breast tumors (Munoz-de-Toro et al., 2005; Tharp et al., 2012; Mandrup et al., 2016). In women, BPA serum concentrations positively correlate with higher breast density, a well-known risk factor for the subsequent development of breast tumors (Sprague et al., 2013). Exposure to BPA also increases the number of pre-cancerous mammary lesions and breast carcinomas and promotes metastasis (Murray et al., 2007; Jenkins et al., 2011).

BPA interacts with intracellular estrogen receptors (ERα, ERβ) and ERRγ (Takayanagi et al., 2006; Delfosse et al., 2012; Liu et al., 2012). It also modulates the activity of other intracellular receptors, such as AR, PR, PPARγ, RXRs, PXR, and TR enhancing cell proliferation and migration (reviewed in Acconcia et al., 2015). However, it acts differently than estrogens on mammary gland (Speroni et al., 2017). In fact, aside from these genomic processes, BPA also acts *via* non-genomic and ER-independent mechanisms through the regulation of intracellular signaling pathways. In breast cancer cells, BPA has been shown to activate ERK (Dong et al., 2011; Song et al., 2015), EGFR (Sauer et al., 2017), FAK, and Src (Castillo et al., 2016), bind to small GTP binding proteins (Schopel et al., 2016), modulate the phosphatidylinositol 3-kinase (PI3-K)/Akt signaling pathway (Goodson et al., 2011), and down-regulate PTEN expression (Wang et al., 2014). These signaling pathways may be activated through binding of BPA to membrane receptors, such as GPR30 (Thomas and Dong, 2006; Dong et al., 2011) or through metalloprotease-mediated shedding of EGFR ligands, leading to EGFR activation (Sauer et al., 2017; Urriola-Munoz et al., 2017). Nowadays, many mechanisms of action have been reported for BPA. However, the association between activated signaling pathways and considered end-points are still unclear.

Protein kinase D1 (PKD1), formerly called PKCµ, is a serine/threonine kinase, expressed in most tissues, that belongs to the Ca^2+^/calmodulin-dependent protein kinase (CAMPK) superfamily (Rozengurt et al., 1995). PKD1 activation requires either phosphorylation by novel protein kinase C (PKC) of two serine residues (S738/742) localized within the activation loop of its catalytic core, or PKC-independent phosphorylation through autophosphorylation of its carboxy-terminal serine residue (S910) (Steinberg, 2012). PKD1 is involved in numerous biological functions, such as cell proliferation, differentiation, apoptosis, invasion, and motility (reviewed in (Sundram et al., 2011) and plays a crucial role in cancer (reviewed in Youssef and Ricort, 2019). We previously demonstrated that PKD1 overexpression potentiates *in vivo* tumor growth of the MCF-7 adenocarcinoma-derived cell line, and regulates cell growth (Karam et al., 2012; Karam et al., 2014). Moreover, we identified PKD1 as a poor prognostic factor in the whole breast cancer population and in the triple-negative breast cancer (TNBC) subtype specifically (Spasojevic et al., 2018). Therefore, due to its crucial role in breast tumor growth and development, we asked in this study whether PKD1 may be a molecular target of BPA.

## Materials and Methods

### Antibodies and Materials

Anti-PKD1 (1/1,000), anti-phospho-S910-PKD1 (1/1,000), anti-phospho-S738/742-PKD1 (1/1,000), and anti-ERα (1/2,000) were purchased from Cell Signaling (Danvers, MA); anti-actin (1/1,000) and anti-GAPDH (1/2,000) from Santa Cruz Biotechnology (Santa Cruz, CA). The horseradish peroxidase-conjugated secondary antibodies used were goat anti-rabbit IgG (1/2,000; Dako, Glostrup, Denmark) and goat anti-mouse IgG (1/5,000; Rockland, Gilbertsville, PA). PRKD1-targeting (#5587) and control siRNAs were purchased from GE Healthcare-Dharmacon (Velizy-Villacoublay, France), Gö6976 and Gö6983 from Calbiochem (Darmstadt, Germany), MTT from Sigma-Aldrich (St. Louis, MO) and BPA (purity 97%+) from Alfa Aesar (Haverhill, MA).

### Tumorigenicity Assay in Athymic Nude Mice

Thirty 8-week old athymic female Swiss nude (*Foxn1^nu/nu^*) mice were purchased from Janvier Labs (Le Genest-Saint-Isle, France) and bred in our animal house for the tumorigenicity assay. All animals were fed *ad libitum* and maintained in accordance with the guidelines for the care and use of laboratory animals of the French Ministry of Agriculture (A-75-06-12). All animals were treated humanely and with regard for alleviation of suffering. Cages and bottles made of polypropylene were used to avoid any BPA contamination. Mice were provided a phytoestrogens and pesticides-free diet containing 16.1% protein, 3.1% fat, and 60.4% carbohydrate (SAFE A04, Safe, Augy, France).

One week after their arrival, mice were randomly allocated to the control (n = 15) or BPA (n = 15) group. They were orally administered either vehicle (ethanol) or 5 µg/kg body weight/day BPA in their drinking water (corresponding to 0.001% ethanol in each water bottle whatever the condition). Treatments were carried out from two weeks before cell injections until day 60 after injection.

Exponentially growing and subconfluent cells (1.2 × 10^7^) were resuspended in 100 μL PBS and injected subcutaneously into the right flank of the mice. Tumors were monitored weekly after inoculation and their volume, in mm^3^, was estimated from the length (L) and width (W) of the tumors using the formula (L × W^2^)/2. Tumors were measured *via* calipers by the same person to avoid significant intra- and inter-personal variation.

### Cell Culture

MCF-7 cells (ATCC) were grown in DMEM-Glutamax^®^ medium (Invitrogen-Life Technologies, Cergy-Pontoise, France) supplemented with 10% fetal bovine serum (FBS), 100 units/mL^−1^ penicillin and 100 mg/mL^−1^ streptomycin. One mg/mL^−1^ G418 (Calbiochem, Darmstadt, Germany) was added to the medium of MCF-7 cells stably overexpressing PKD1 (clone P) or not (clone C). As initially described in (Karam et al., 2012), clones P and C were stable transfected with pcDNA-3-PKD1 or pcDNA-3, respectively. Prior to experiments, cells were cultured for 24 h in estrogen-free medium consisting of phenol red-free DMEM supplemented with 10% charcoal-treated FBS, 1% sodium pyruvate, 1% L-glutamine, and 100 units/mL^−1^ penicillin and 100 mg/mL^−1^ streptomycin. Charcoal treatment was used to remove non-polar material such as lipophilic molecules such as steroid hormones without little effect on salts, glucose, or amino acids. It thus allows the design of a culture medium favorable in studying processes influenced by steroid hormones such as estrogen, and endocrine disruptors such as BPA. Cells were tested for mycoplasma (Venor^®^ *GeM* Advance, Minerva Biolabs, Germany) every three months and prior any injection to nude mice.

### MTT Assay

MCF-7 cells were seeded in quadruplicate into 96-well plates at a density of 1.5 × 10^3^ cells/well and allowed to adhere overnight. The next day, the culture medium was replaced by estrogen-free medium supplemented with 1% charcoal-treated FBS and cells were incubated for 72 h with or without BPA (10^−6^ to 10^−10^ M), 0.5 µM Gö6976, 0.5 µM Gö6983, or vehicle (DMSO). At the end of the incubation period, cells were incubated for 4 h with mL MTT · mL^−1^ MTT. The medium was then gently removed, 200 μL DMSO added, and the absorbance measured at 570 nm using a plate reader (BMG Labtech).

### siRNA Transfection

siRNA transfection was performed according to the manufacturer’s protocol (Santa Cruz). Briefly, 3 × 10^5^ cells were seeded per well in 2 mL antibiotic-free DMEM supplemented with 10% FBS. After 24 h, 1 µg siRNA and 8 µL siRNA transfection reagent, each diluted in 100 µL siRNA transfection medium, were combined, incubated for 45 min at room temperature, and applied to the cells in a final volume of 1 mL siRNA transfection medium. After a 7-h incubation, cells were incubated in estrogen-free medium supplemented with 10% charcoal-treated FBS for an additional 18 h before analysis.

### Clonogenic Assay

MCF-7 cells, seeded in duplicate in six-well plates at a density of 400 cells/well, were incubated for 14 days in estrogen-free medium containing 10^−6^ to 10^−10^ M BPA, 0.5 µM Gö6976, 0.5 µM Gö6983, or vehicle (DMSO). At the end of the incubation period, the colonies were washed twice with PBS, fixed with 60% ice-cold ethanol for 10 min at −20°C, and stained with 0.5% crystal violet for 1 h at room temperature. Colonies consisting of at least 50 cells were counted and their area determined using Image J software.

### Anchorage-Independent Growth Assay

MCF-7 cells (1.10^4^) were resuspended in 2.5 mL methylcellulose (0.8%) prepared in estrogen-free medium containing 10^−6^ to 10^−10^ M BPA, 0.5 µM Gö6976, 0.5 µM Gö6983, or vehicle (DMSO). Cells were plated in uncoated 35-mm culture dishes and incubated for three to five weeks. Plates were then photographed and macroscopic colonies counted using an optical microscope (Zeiss Leica, Axiostar).

### Western Immunoblotting

Cells were lysed for 20 min at 4°C in 50 mM Tris-HCl pH 7.4, 150 mM NaCl, 1 mM EDTA, 100 mM sodium fluoride, 10 mM tetra-sodium diphosphate decahydrate, 2 mM sodium orthovanadate, 1 mM phenylmethylsulfonylfluoride, 10 µg/mL aprotinin, and 1% Nonidet P-40. Lysates were clarified by centrifugation at 18,000 *g* for 10 min at 4°C. The protein concentration was determined with the BC Assay Protein Quantification kit (Interchim, Montluçon, France) using bovine serum albumin as standard. Total proteins (40 to 80 µg) were separated by SDS-PAGE and transferred onto nitrocellulose membranes. Membranes were incubated overnight at 4°C with the primary specific antibodies, then incubated for 1 h at room temperature with the peroxidase-conjugated secondary antibodies and revealed by enhanced chemiluminescence (Amersham, GE Healthcare, UK).

### Statistical Analysis

For *in vitro* experiments, the statistical significance of differences between experimental groups was determined with the Mann Whitney test using Prism 5.03 software for Windows (GraphPad Software, San Diego, CA). Differences between values were considered to be significant when p ≤ 0.05 (*) and highly significant when p ≤ 0.01 (**) or p ≤ 0.001 (***).

## Results

### BPA Specifically Stimulates MCF-7 Cells Proliferation

**Figure 1:**
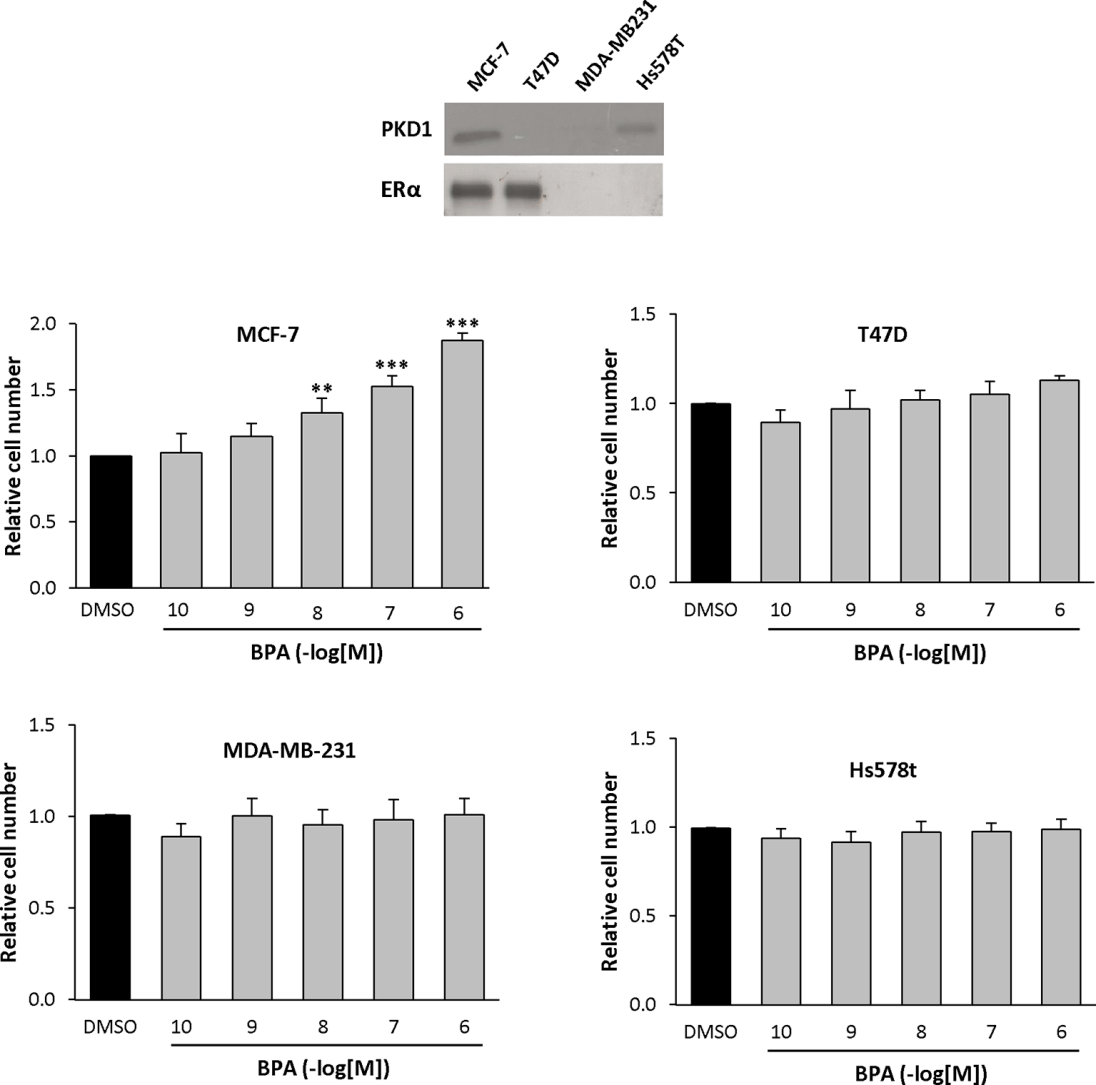
BPA differentially modulates cell proliferation of breast cancer cell lines. MCF-7, T47D, Hs578t, and MDA-MB-231 cells were incubated in the presence of DMSO (vehicle) or increasing concentrations of BPA (10^−10^ to 10^−6^ M). Viable cells were identified over 72 h using MTT assay. The results are presented as the mean ± SD of at least three independent experiments and are expressed relative to control (vehicle-treated) cells.**p ≤ 0.01 and ***p ≤ 0.001 versus vehicle-treated cells. Upper: PKD1 and ERα proteins detected by Western blotting in each cell line.

BPA was described to regulate breast cancer cell proliferation through ERα-dependent and -independent mechanisms (Jedeon et al., 2014). Moreover, we demonstrated that PKD1 expression levels affect breast cancer cell proliferation *in vitro* and *in vivo* (Karam et al., 2012; Karam et al., 2014). Therefore, we asked whether BPA may specifically modulate the proliferation of four breast cancer cell lines that differently express ERα and PKD1. MCF-7 (ERα+, PKD1+), T47D (ERα+, PKD1−), Hs578t (ERα−, PKD1+), and MDA-MB231 (ERα−, PKD1−) cells (insert, **Figure 1**) were treated with different concentrations of BPA (10^−10^ to 10^−6^ M) and analyzed for cell number using MTT assay as indicated in *Materials and Methods*. BPA dose-dependently increased MCF-7 cell number (**Figure 1**) with a significant effect (1.33-fold ± 0.11) from as low a concentration as 10^−8^ M and a maximal effect (1.88-fold ± 0.15) at 10^−6^ M. Neither MDA-MB-231, nor T47D, nor Hs578t cells responded to BPA for cell proliferation. These results suggest that BPA-stimulated MCF-7 cell growth depends upon PKD1 expression.

### BPA Stimulates Cell Growth Through a PKD1-Dependent Mechanism

**Figure 2:**
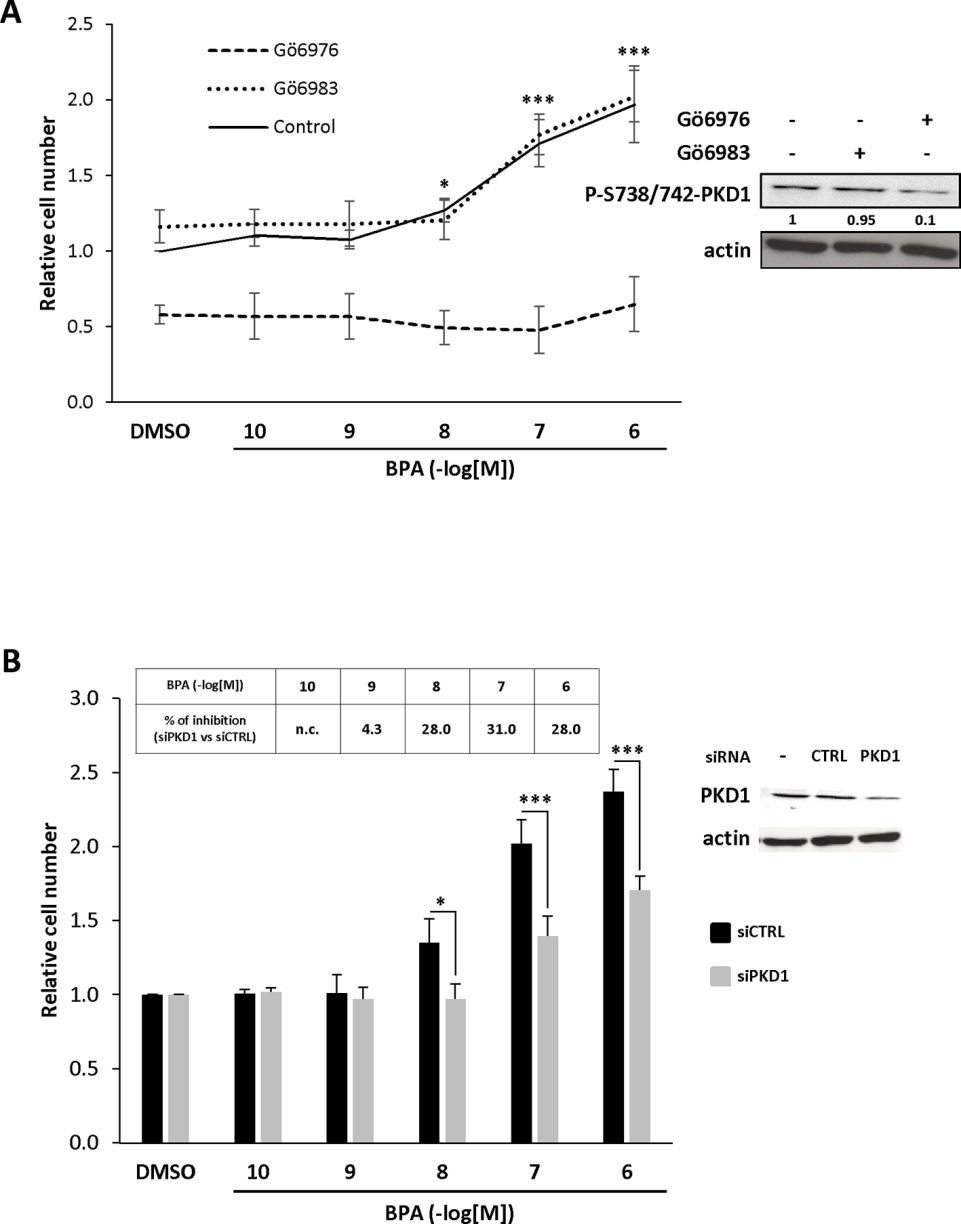
BPA increases MCF-7 proliferation through a PKD1-dependent signaling pathway. **(A)** MCF-7 cells were incubated in the presence of DMSO (vehicle) or increasing concentrations of BPA (10^−10^ to 10^−6^ M) with or without 0.5 μM Gö6976 or 0.5 μM Gö6983. Viable cells were identified over 72 h by MTT assay. The results are presented as the mean ± SD of four independent experiments. *p ≤ 0.05 and ***p ≤ 0.001 *versus* vehicle-treated cells. Left: Western blot detection of phospho-S738/742-PKD1 and actin in MCF-7 cells treated for 72 h with or without 0.5 μM Gö6976 or 0.5 μM Gö6983. Values presented under the top autoradiogram represent the quantitative analysis of each band as the fold increase relative to untreated cells, normalized against the actin signal. **(B)** MCF-7 cells were transfected with PKD1 (siPKD1) or control (siCTRL) siRNAs. The next day, cells were cultured in the presence of DMSO or increasing concentrations of BPA (10^−10^ to 10^−6^ M) and cell survival analyzed 72 h later using MTT assays as described in panel **(A)**. The results are presented as the mean ± SEM of three independent experiments. *p ≤ 0.05 and ***p ≤ 0.001. The table presents the percentage inhibition of BPA-stimulated cell proliferation between siPKD1- and siCTRL-transfected cells, calculated for each BPA concentration (n.c. not calculated). Upper right: Western blot detection of PKD1 and actin in MCF-7 cells transfected with PKD1 or control (CTRL) siRNAs.

To assess whether PKD1 plays a role in BPA-induced mitogenic effects, MCF-7 cells were incubated with two different inhibitors, the indolocarbazole Gö6976, which inhibits conventional PKCs (PKCα, PKCβ) and PKD1 with an IC50 of approximately 20 nM, and the bisindolylmaleimide Gö6983, which very poorly inhibits PKD1 kinase activity (IC50 = 20 μM), whereas it strongly inhibits PKCα and PKCβ kinase activity (IC50 = 7 nM). Phosphorylation of PKD1 on S738/742 residues was strongly inhibited by 0.5 µM Gö6976, but not at all affected by 0.5 µM Gö6983 treatment (insert, **Figure 2A**), as expected. We then studied the effect of these two inhibitors on BPA-induced MCF-7 cell proliferation. Gö6983 treatment did not affect the response of MCF-7 cells to BPA (**Figure 2A**). In contrast, Gö6976 treatment completely suppressed the ability of BPA to stimulate cell proliferation, even at the highest concentration of 1 µM (**Figure 2A**). To further strengthen these results, we also inhibited PKD1 by transfecting cells with a siRNA that specifically targets PKD1 mRNA (siPKD1). PKD1 expression was almost completely suppressed compared to cells transfected with a control scrambled siRNA (siCTRL), which had no effect (insert, **Figure 2B**). BPA-induced cell proliferation was reduced by 28–31% in cells transfected with PKD1-targeting siRNA, whereas control siRNA (siCTRL) had no detectable effect (**Figure 2B**). Collectively, these results show that PKD1 plays a crucial role in BPA-stimulated MCF-7 cell proliferation.

### PKD1 Contributes to BPA-Induced Clonogenicity

**Figure 3:**
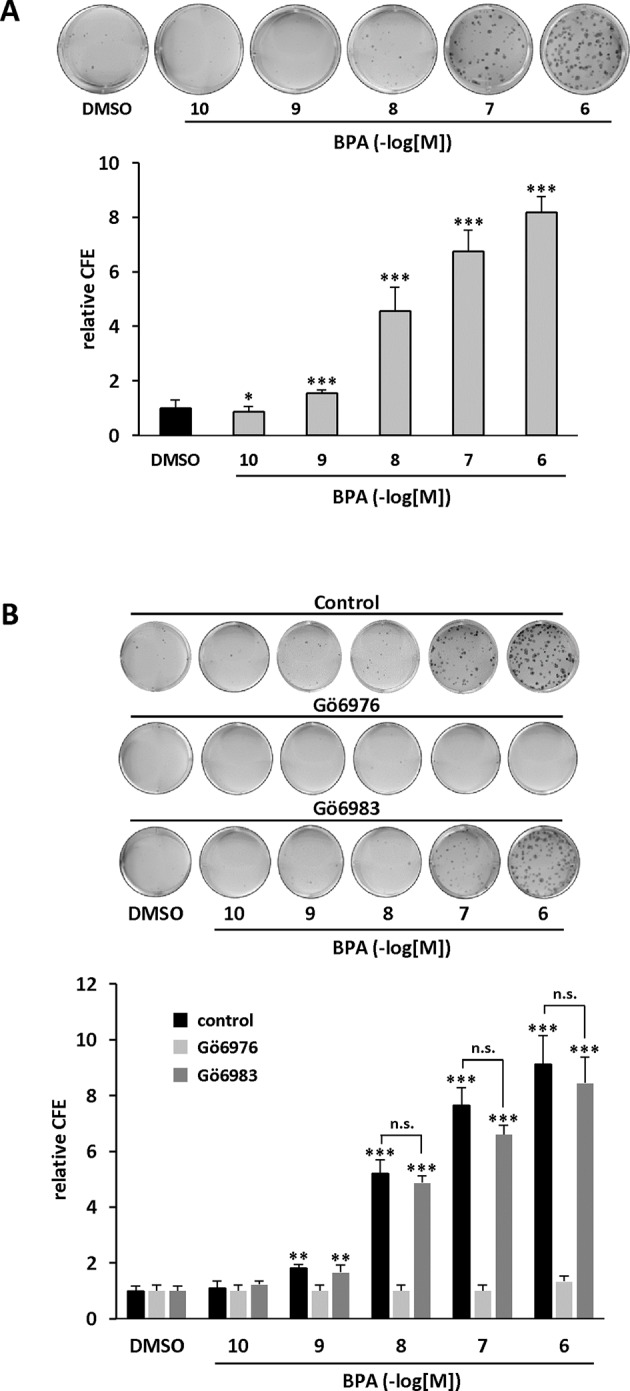
BPA stimulates MCF-7 clonogenicity through a PKD1-dependent signaling pathway. **(A)** MCF-7 cells, seeded at a density of 400 cells/well, were incubated in the presence of DMSO (vehicle) or increasing concentrations of BPA (10^−10^ to 10^−6^ M). After 10 days, the clones were photographed (top) and their number determined (bottom). The results are presented as the mean ± SD of three independent experiments and are expressed as the colony forming efficiency (CFE: (number of clones formed/number of cells seeded) × 100). *p ≤ 0.05 and ***p ≤ 0.001 *versus* vehicle-treated cells. **(B)** Same experiment as in panel **(A)**, in which cells were also treated with or without 0.5 μM Gö6976 or 0.5 μM Gö6983. The results are presented as the mean ± SD of three independent experiments. **p ≤ 0.01 and ***p ≤ 0.001 *versus* control vehicle-treated cells. ns, not significant.

**Figure 4:**
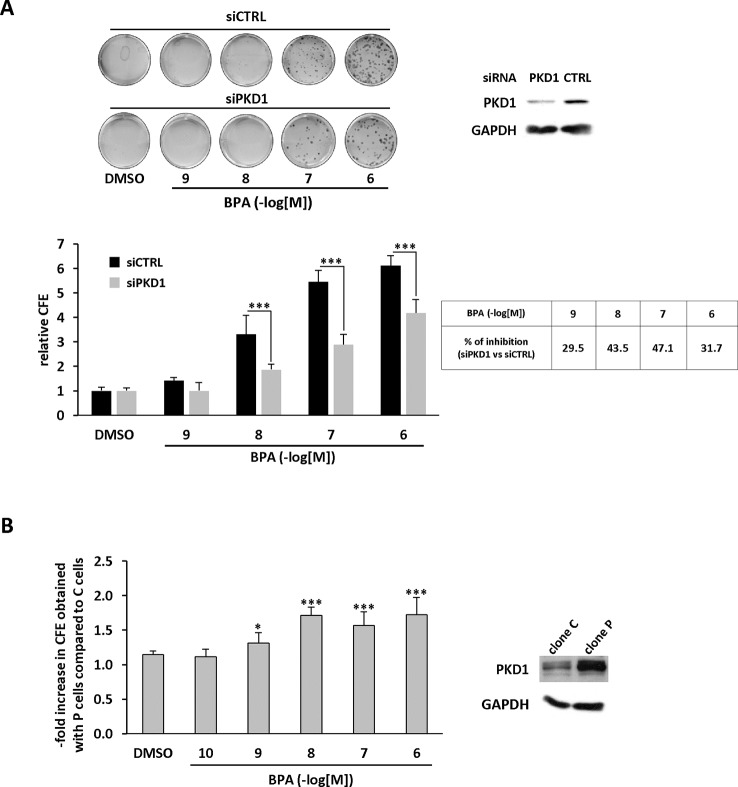
BPA stimulates MCF-7 clonogenicity through a PKD1-dependent signaling pathway. **(A)** MCF-7 cells were transfected with PKD1 (siPKD1) or control (siCTRL) siRNAs. The next day, cells seeded at a density of 400 cells/well, were incubated in the presence of DMSO (vehicle) or increasing concentrations of BPA (10^−9^ to 10^−6^ M). After 10 days, the clones were photographed (top) and their number determined (bottom). The results are presented as the mean ± SD of three independent experiments and are expressed as the colony forming efficiency (CFE: (number of clones formed/number of cells seeded) × 100). The results are presented as the mean ± SD of three independent experiments. ***p ≤ 0.001. The table presents the percentage of inhibition of BPA-stimulated cell proliferation between PKD1- and CTRL-siRNA-transfected cells. Upper right: Western blot detection of PKD1 and GAPDH in MCF-7 cells transfected with PKD1 or control (CTRL) siRNAs. **(B)** MCF-7 overexpressing PKD1 (clone P), or not (clone C), were seeded and treated as described in panel **(A)**. Results are expressed as the fold increase in CFE obtained with PKD1-overexpressing cells (clone P) over control cells (clone C), for each treatment condition. *p ≤ 0.05 and ***p ≤ 0.001 *versus* clone C. Right: PKD1 protein expression in MCF-7 cells overexpressing PKD1 (clone P), or not (clone C). ***p ≤ 0.001 *versus* control vehicle-treated cells.

We further characterized the role of PKD1 in BPA response by investigating the role of PKD1 on BPA-induced MCF-7 clonogenicity. BPA dose-dependently stimulated MCF-7 colony formation relative to vehicle-treated cells, with a significant effect observed from 10^−9^ M to 10^−6^ M (**Figure 3A**). Interestingly, cell clones were not only more numerous, but also significantly larger. Gö6976 completely blocked BPA-induced clone formation, whereas Gö6983 had no significant effect (**Figure 3B**). Moreover, PKD1-targeting siRNA (siPKD1) strongly inhibited (by 32 to 47%) BPA-induced colony formation of MCF-7 cells (**Figure 4A**), whereas the control siRNA (siCTRL) had no effect. PKD1 overexpression increased cell sensitivity to BPA, as PKD1-overexpressing MCF-7 cells (clone P) formed more colonies than control cells (clone C) (**Figure 4B**). Overall, these data thus show that BPA increases proliferation and survival of low density-seeded MCF-7 cells by a PKD1-dependent mechanism.

### PKD1 Contributes to BPA-Induced Anchorage-Independent Growth Stimulation

**Figure 5:**
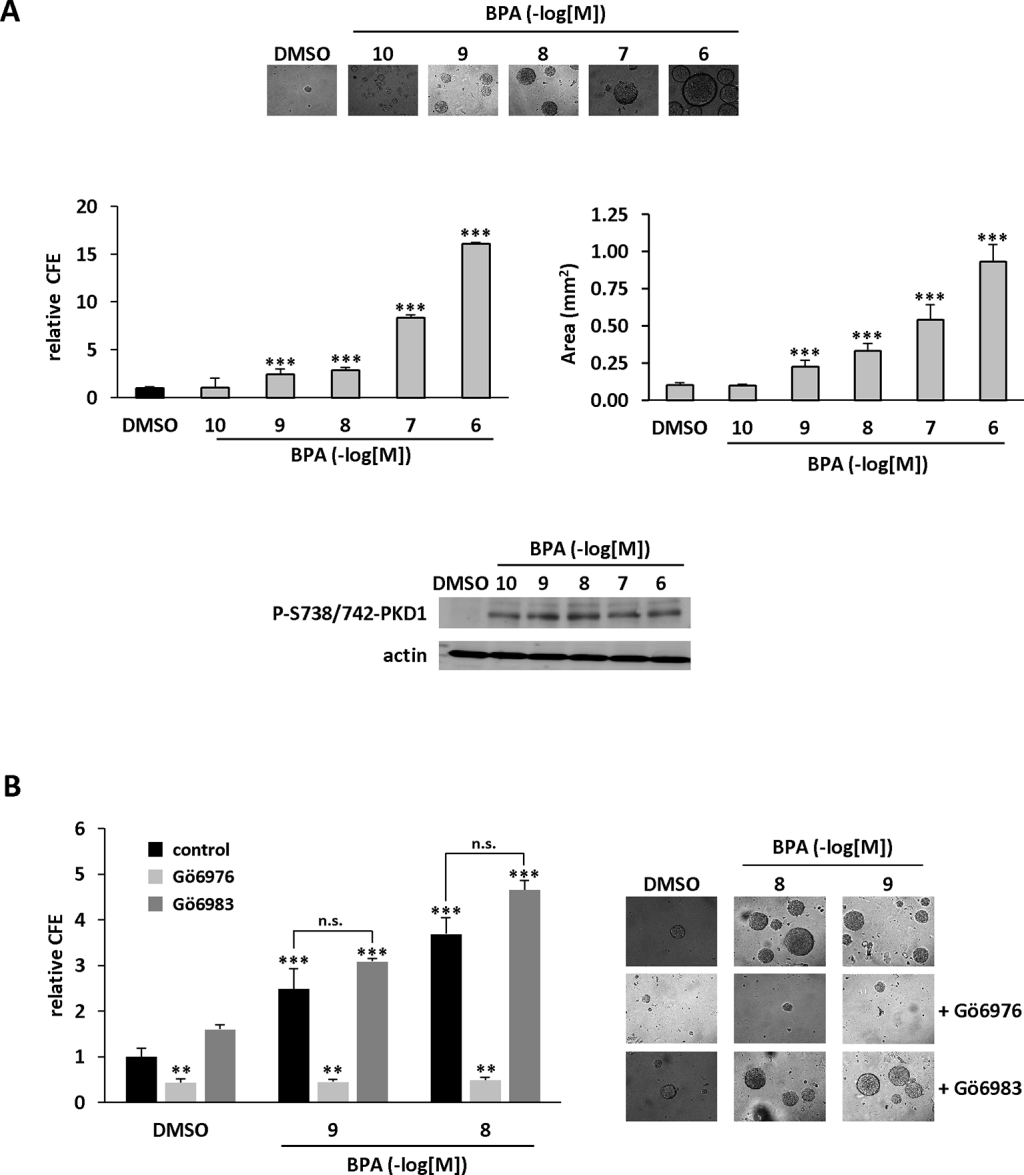
BPA stimulates MCF-7 anchorage-independent growth through a PKD1-dependent signaling pathway. **(A)** MCF-7 cells were seeded at a density of 10,000 cells/well in semi-solid medium in the presence of DMSO (vehicle) or increasing concentrations of BPA (10^−10^ to 10^−6^ M). After three weeks, the clones were photographed (top), counted (middle left), and their surface area calculated (middle right, in mm^2^). The results are presented as the mean ± SD of three independent experiments. The percentage of colony forming efficiency (% CFE = (number of clones formed/number of cells seeded)*100) is expressed as the fold increase over vehicle-treated cells. ***p ≤ 0.001. Bottom: Western blot detection of phospho-738/742-PKD1 and actin in MCF-7 clones collected at the end of the experiment. **(B)** Same experiment as in panel **(A)**, in which cells were also treated with or without 0.5 μM Gö6976 or 0.5 μM Gö6983. The results are presented as the mean ± SD of three independent experiments. **p ≤ 0.01 and ***p ≤ 0.001 *versus* control vehicle-treated cells. ns, not significant.

**Figure 6:**
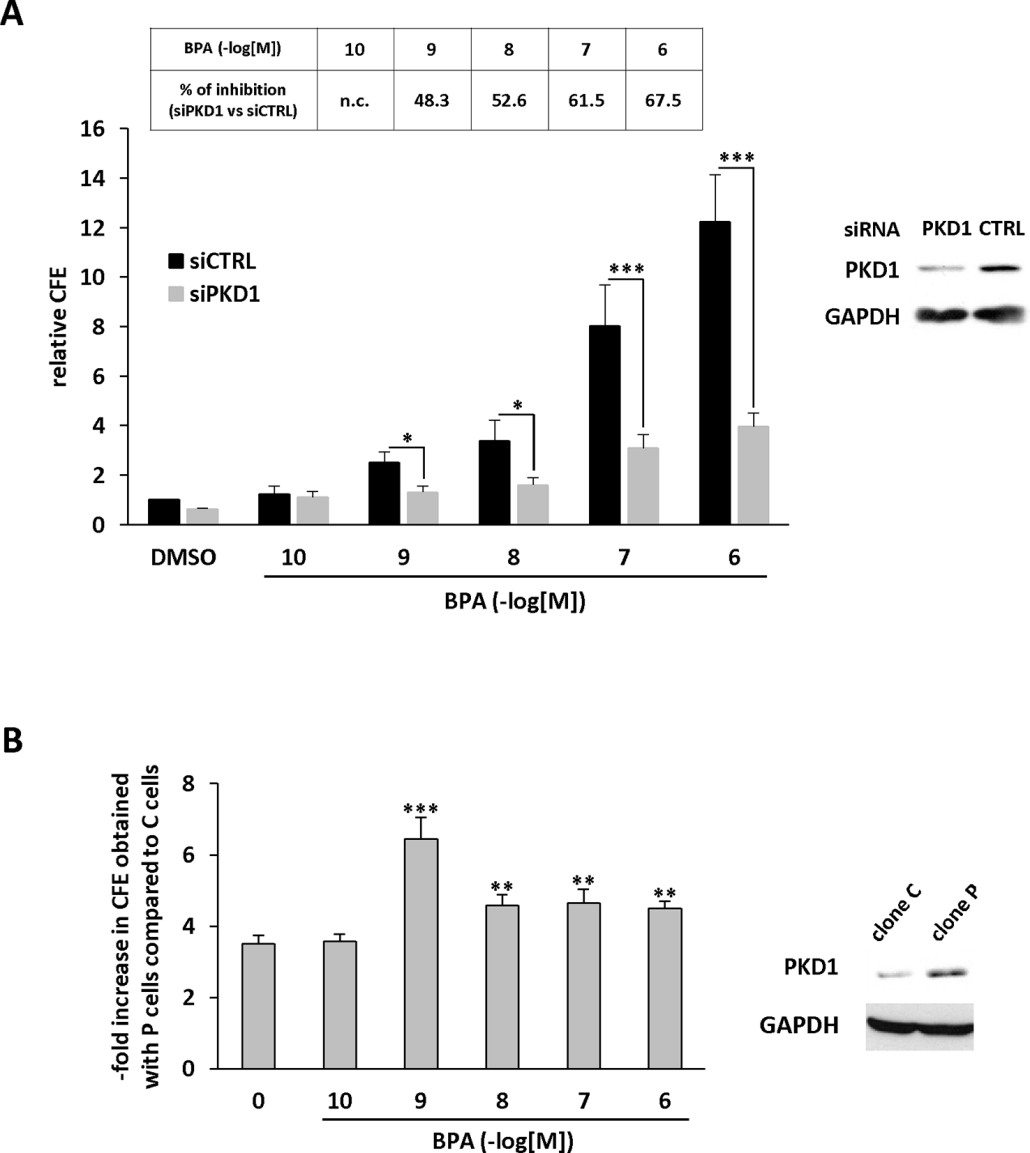
BPA stimulates MCF-7 anchorage-independent growth through a PKD1-dependent signaling pathway. **(A)** MCF-7 cells were transfected with PKD1 (siPKD1) or control (siCTRL) siRNAs. The next day, cells were seeded at a density of 10,000 cells/well in semi-solid medium in the presence of DMSO (vehicle) or increasing concentrations of BPA (10^−10^ to 10^−6^ M). After three weeks, the clones were counted. The results are presented as the mean ± SD of three independent experiments and are expressed as the percentage of CFE expressed as the fold increase over vehicle-treated cells. The table presents the percentage of inhibition of BPA-stimulated CFE formation between siPKD1- and siControl-transfected cells (n.c. not calculated). *p ≤ 0.05 and ***p ≤ 0.001 *versus* siControl-transfected cells. Right: Western blot detection of PKD1 and GAPDH in MCF-7 cells transfected with PKD1 or control (CTRL) siRNAs. **(B)** MCF-7 overexpressing PKD1 (clone P), or not (clone C), were seeded and treated as described in panel **(A)**. Results are expressed as the fold increase of CFE obtained with PKD1-overexpressing cells (clone P) over control cells (clone C), for each treatment condition. **p ≤ 0.01 and ***p ≤ 0.001 *versus* clone C.

Anchorage-independent growth is a key aspect of the tumor phenotype, particularly with respect to metastatic potential. Thus, we determined whether BPA modulates colony formation of methylcellulose-cultured MCF-7 cells and whether PKD1 plays a role in this process. BPA dose-dependently stimulated colony formation (**Figure 5A**), with a significant effect (2.43-fold ± 0.61) from 10^−9^ M and a maximal effect (16.04-fold ± 0.08) at 10^−6^ M. Moreover, BPA not only increased the number of clones, but also their size, as their mean area increased from 0.01 ± 0.02 mm^2^ under vehicle-treatment to 0.93 ± 0.12 mm^2^ when treated with 10^−6^ M BPA. Interestingly, BPA-treated MCF-7 cells displayed a higher level of PKD1 phosphorylation on serine 738/742 residues than vehicle-treated cells, regardless of the concentration used (**Figure 5A**). Gö6976, but not Gö6983 (**Figure 5B**), and PKD1-targeting siRNA (siPKD1), but not a control siRNA (siCTRL) (**Figure 6A**), strongly impaired BPA-induced anchorage-independent growth of MCF-7 cells. In contrast, overexpression of PKD1 increased the response of MCF-7 cells to BPA (**Figure 6B**). Altogether, these results demonstrate that PKD1 is a key determinant in the ability of BPA to stimulate *in vitro* 3D colony formation of MCF-7 cells.

### BPA Stimulates the Phosphorylation of PKD1

**Figure 7:**
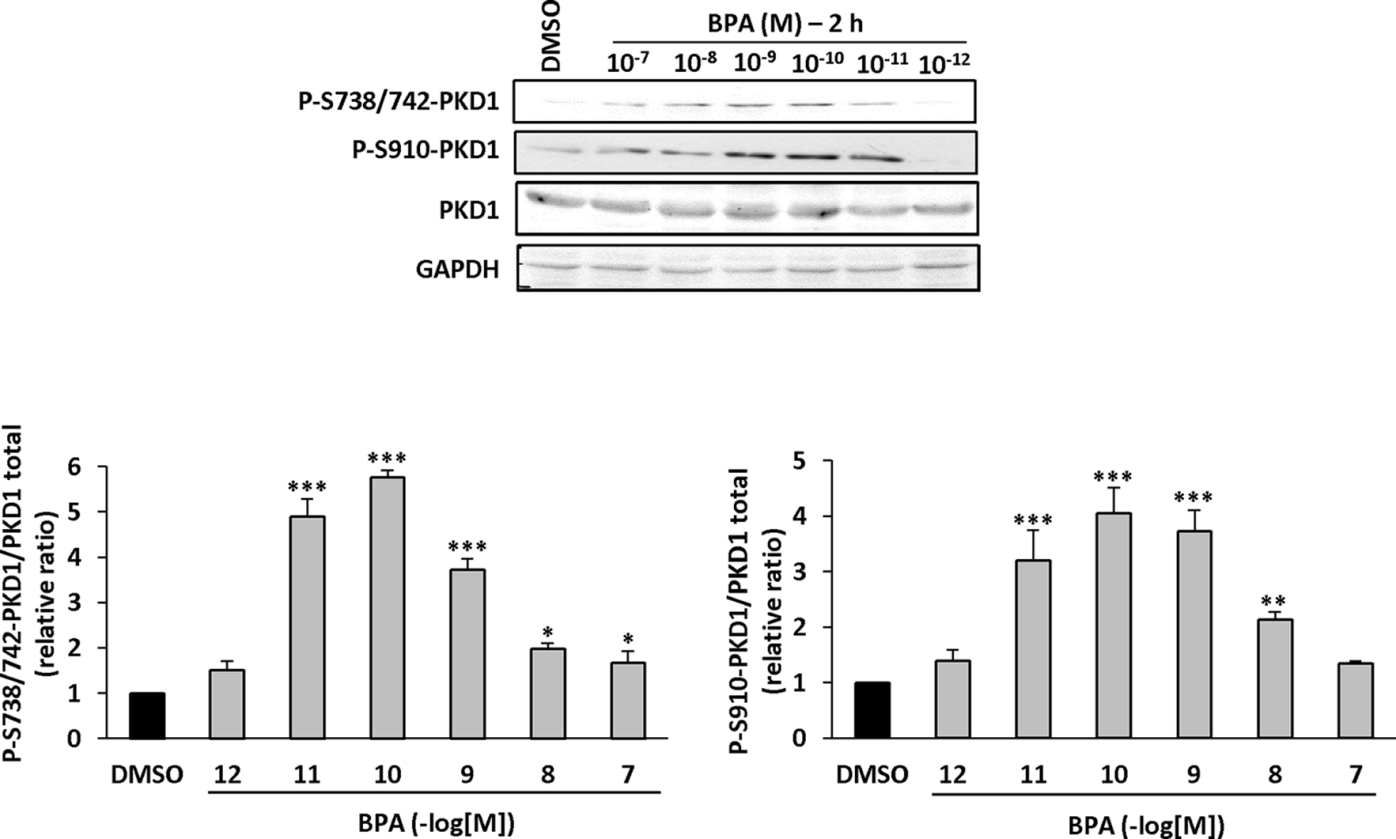
BPA induces PKD1 phosphorylation in MCF-7 cells. MCF-7 cells were incubated with or without increasing concentrations of BPA (10^−10^ to 10^−6^ M) for 2 h. At the end of the incubation, cells were lysed and equal amount of proteins separated by SDS-PAGE, transferred to nitrocellulose, and detected with anti-phospho-S738/742-PKD1, anti-phospho-S910-PKD1, anti-PKD1, or anti-GAPDH antibodies. The autoradiograms presented are from typical experiments. Bars represent the quantitative analysis of phosphorylated PKD1 under each set of conditions, corrected for background and normalized to PKD1 and GAPDH signals, and expressed as the fold increase relative to vehicle-treated cells. The results are presented as the mean ± SD of three independent experiments. *p ≤ 0.05, **p ≤ 0.01, and ***p ≤ 0.001 *versus* vehicle-treated cells.

**Figure 8:**
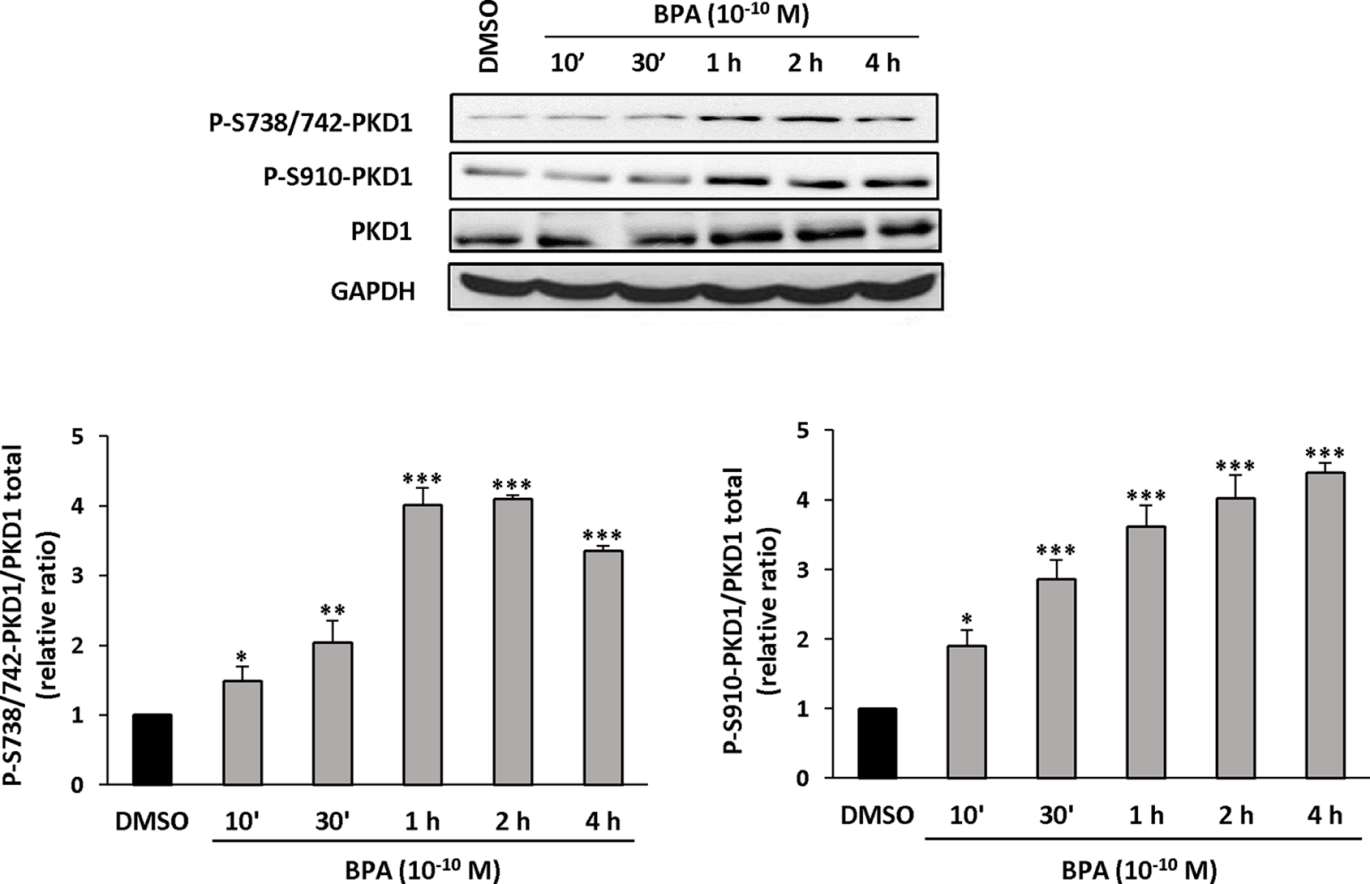
BPA induces PKD1 phosphorylation in MCF-7 cells. MCF-7 cells were incubated for different periods of time (10 min to 4 h) with or without BPA (10^−10^ M). At the end of the incubation, cells were lysed and equal amount of proteins separated by SDS-PAGE, transferred to nitrocellulose, and detected with anti-phospho-S738/742-PKD1, anti-phospho-S910-PKD1, anti-PKD1, or anti-GAPDH antibodies. The autoradiograms presented are from typical experiments. Bars represent the quantitative analysis of phosphorylated PKD1 under each set of conditions, corrected for background and normalized to PKD1 and GAPDH signals, and expressed as the fold increase relative to vehicle-treated cells. The results are presented as the mean ± SD of three independent experiments. *p ≤ 0.05, **p ≤ 0.01, and ***p ≤ 0.001 *versus* vehicle-treated cells.

**Figure 9:**
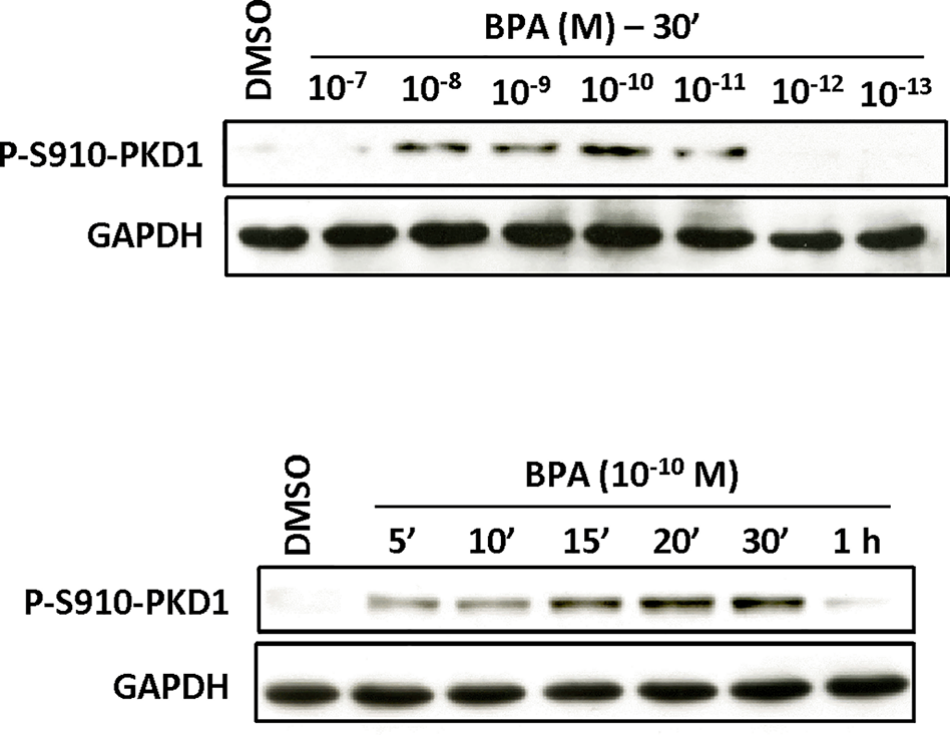
BPA induces PKD1 phosphorylation in Hs578t cells. Hs578t cells were incubated (top) with or without different concentrations (10^−7^ to 10^−13^ M) of BPA for 30 min or (bottom) for different periods of time (5 min to 1 h) with or without BPA (10^−10^ M). At the end of the incubation, cells were lysed and equal amount of proteins separated by SDS-PAGE, transferred to nitrocellulose, and detected with anti-phospho-S910-PKD1, or anti-GAPDH antibodies as described in *Materials and Methods*. The autoradiograms presented are from typical experiments.

Since PKD1 appears to play a crucial role in the response of MCF-7 cells to BPA, we next determined whether and how BPA modulates PKD1 activity. MCF-7 cells were treated with or without different concentrations of BPA (10^−12^ to 10^−7^ M) and the phosphorylation state of PKD1 analyzed. BPA (10^−11^ to 10^−8^ M) dose-dependently stimulated phosphorylation of PKD1 on serine 738/742 and serine 910 residues with a maximal effect from 10^−9^ to 10^−11^ M (**Figure 7**). This occurred without any change in PKD1 total expression (**Figure 7**). Time-course analysis showed that 10^−10^ M BPA rapidly stimulated serine 738/742 and serine 910 phosphorylation of PKD1 with a maximal effect after 1 h of BPA treatment, which remained mostly stable for up to 2 to 4 h (**Figure 8**). BPA did not affect the level of PKD1 expression at any time point analyzed, as previously verified (**Figure 8**). BPA-induced PKD1 phosphorylation also occurred in Hs578t cells with a dose-response similar to that observed in MCF-7 cells (**Figure 9**). However, time-course analysis showed that, compared to MCF-7 cells, BPA-induced PKD1 phosphorylation occurred more rapidly (maximal effect observed after 20–30 min of BPA treatment) and was more transient (phosphorylation levels returned to basal values after 1 h of BPA treatment) in Hs578t (**Figure 9**).

### PKD1 Affects BPA-Induced Tumor Growth *In Vivo*

**Figure 10:**
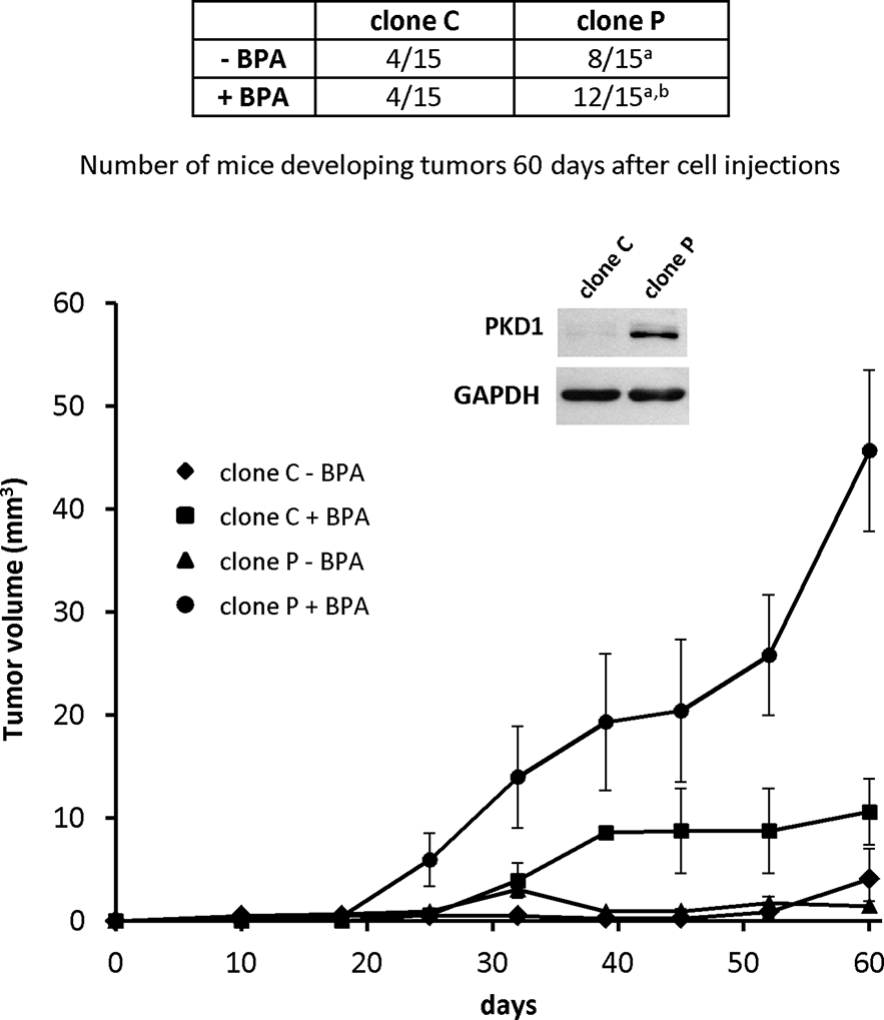
PKD1 increases BPA-induced breast tumor tumorigenicity. MCF-7 cells (1.2 × 10^7^) overexpressing PKD1 (clone P), or not (clone C), were subcutaneously injected into *Foxn1^nu/nu^* nude mice orally exposed (+BPA) or not (- BPA) to BPA (5 µg/kg body weight per day). Fifteen mice were used per condition tested. The table shows the number of mice carrying tumors at the end of the experiment (day 60 post-injection), whereas the graph shows the mean tumor volume (in mm^3^ ± SEM) measured each week. Insert: PKD1 protein expression in MCF-7 cells overexpressing PKD1 (clone P), or not (clone C). p ≤ 0.001 *versus* MCF-7 cells not overexpressing PKD1 (clone C)^a^ and p ≤ 0.01 *versus* mice not exposed to BPA^b^.

As PKD1 contributes to BPA-induced MCF-7 cells proliferation *in vitro*, we explored whether PKD1 may sensitize cancer cells to BPA *in vivo*. Nude mice were subcutaneously injected with 1.10^7^ cells overexpressing PKD1 (clone P), or not (clone C), and orally exposed, or not, to 5 µg/kg/day BPA without any estrogen supplementation. Sixty days after cell injections, mice injected with PKD1-overexpressing cells (clone P) developed more tumors (8 of 15) than mice injected with control (clone C) cells (4 of 15), in accordance with our previous results (**Figure 10**) (Karam et al., 2012). Exposure to BPA appeared to have no effect on the number of mice developing tumors when they were injected with control cells (4 of 15). However, exposure to BPA markedly increased the number of mice developing tumors when injected with PKD1 overexpressing cells (12 out of 15). BPA also significantly increased the mean tumor volume, regardless of the cells injected, but more importantly when cells overexpress PKD1 (**Figure 10**). Indeed, tumor volumes of BPA-free mice injected with MCF-7 cells overexpressing or not PKD1 were 1.44 ± 0.05 mm^3^ and 4.09 ± 2.89 mm^3^ after 60 days, respectively. In the presence of BPA, tumor volumes were significantly higher, 45.67 ± 20.74 mm^3^ and 10.59 ± 3.20 mm^3^ after 60 days, for cells overexpressing or not PKD1, respectively (**Figure 10**). Overall, these results show that BPA promoted tumor growth *in vivo* and that PKD1 expression levels modulated the cellular response to this endocrine disruptor.

## Discussion

We have identified the serine/threonine kinase PKD1 as a new non-genomic functional target of BPA in breast cancer cells *in vitro*. Moreover, we showed that increasing PKD1 expression levels significantly sensitized breast cancer cells to BPA-induced *in vivo* tumor growth. These results are of crucial importance, because 1) they show that daily exposure of mice to low-dose BPA (5 µg/kg/d), similar to the temporary Tolerable Daily Intake established by EFSA (2015), may promote *in vivo* xenograft breast tumor growth and, more importantly, 2) they identified PKD1 as a key functional target of this process.

PKD1 is ubiquitously expressed (Jaggi et al., 2007), implying many cell types may rapidly respond to BPA. However, cell responses to BPA may vary from one cell type to another as demonstrated in MCF-7 and Hs578t cells. In fact, although BPA stimulates PKD1 phosphorylation in both cell lines, it does not affect the proliferation of Hs578t cells contrary to MCF-7 cells, highlighting the importance of the cellular context for sensitivity to the growth-promoting action of BPA. In addition, PKD1 phosphorylation time-courses are not identical in the two cell models, being more transient in Hs578t than in MCF-7 cells suggesting that a more sustained activation of PKD1 could be necessary to mediate a pro-proliferative message. Among the suspected molecular partners of PKD1, ERα has been largely described as the main intracellular receptor of BPA and although the affinity of BPA for ERα is approximately 1,000-fold lower than that of its natural ligand, E2, existing data support a role for this receptor in most cellular responses to BPA (Jedeon et al., 2014) [review in (Alonso-Magdalena et al., 2012)]. Here, the analysis of four breast cancer cell lines expressing PKD1, or not, and/or ERα revealed that only both the PKD1- and ERα-positive MCF-7 cells responded to BPA. In fact, both TD47 (ERα+, PKD1−) and Hs578t (ERα−, PKD1+) failed to respond to BPA for cell proliferation strongly suggesting that PKD1 and ERα act together to allow an efficient proliferative response to BPA. Therefore, the relationship between PKD1 and ERα in the context of cell response to BPA needs to be further considered, not only in breast tumors, but also in all ERα- and PKD1-expressing tissues, regardless of cell phenotype, tumorigenic or not.

As hypothesized for the cellular context, the level of expression of PKD1 seems also to be determinant for the response to BPA. In fact, BPA-treated mice injected with (control) MCF-7 cells displayed bigger tumors than the vehicle-treated group but the number of mice developing tumors (4 of 15) was unchanged. However, mice injected with PKD1-overexpressing cells and treated with BPA developed more (12/15 vs. 8/15, respectively) and bigger (31.7-fold increase) tumors than their vehicle-treated counterparts. Since we voluntary used a MCF-7 cell model that slightly overexpresses PKD1 in order not to drastically affect the PKD1-dependent signaling pathways, these results suggest 1) that PKD1 seems to be a very sensitive factor for the cell response to BPA and 2) that an increased PKD1 expression drastically affects cell behavior to BPA from a pro-proliferative response to a pro-proliferative and pro-tumorigenic one. These experimental data argue for the importance of PKD1 expression level in tumors that may influence their susceptibility to BPA.

We explored how PKD1 may sensitize cancer cells to BPA by testing whether BPA can increase breast tumor growth by regulating cell survival and/or cell proliferation, in which PKD1 plays a crucial role (Sundram et al., 2011). We demonstrated that BPA increased cell number, anchorage-independent growth, and clonogenicity of MCF-7 cells *in vitro* through PKD1-dependent mechanisms using multiple molecular approaches. The central role of PKD1 is particularly illustrated by its ability to act 1) downstream of a large number of extra- or intracellular stimuli, such as growth factors, peptides, thrombin, bioactive lipids, chemokines, or reactive oxygen species [reviewed in (Roy et al., 2017)], 2) at the crossroad of the diacylglycerol (DAG) and protein kinase C (PKC) family-dependent signaling pathways [reviewed in (Rozengurt, 2011)], and 3) upstream of several targets involved in various functions, such as the regulation of gene expression, DNA synthesis, mitochondrial behavior, vesicular traffic, or secretion [reviewed in (Sundram et al., 2011)]. This property of PKD1 to proceed as an integration point may be also due to its ability to translocate to different specific subcellular compartments in response to stimulating signals (Matthews et al., 2000). These characteristics make PKD1 an ideal candidate to regulate signaling pathways, such as those involved in 2D- or 3D-cell proliferation, or clonogenicity. Therefore, our results showing the major role of PKD1 in BPA-stimulated cell proliferation are not completely surprising, given its important cellular functions, and are in accordance with our previous results demonstrating that PKD1 plays a crucial role in anchorage-dependent and -independent growth *in vitro* and tumor growth *in vivo* (Karam et al., 2012). Moreover, they further support PKD1 as a pro-survival and pro-proliferative protein, as demonstrated in numerous cell models [reviewed in (Youssef and Ricort, 2019)].

We provide direct evidence of the impact of BPA on PKD1 by demonstrating that BPA induced the rapid and dose-dependent phosphorylation of PKD1 at two sites (S738/742 and S910), known to be representative of the activation state of PKD1 (Steinberg, 2012). BPA-stimulated PKD1 phosphorylation is one of the strongest arguments for designating PKD1 as a non-genomic target of BPA and is illustrated in two PKD1-expressing breast cancer cell models. Indeed, short-term BPA-induced PKD1 phosphorylation, in the absence of any changes in PKD1 expression, suggests the existence of short and rapid signaling pathways between putative BPA receptors and PKD1. PKD1 is, among other things, stimulated by ligands acting through G-protein coupled receptors, such as bombesin, vasopressin, endothelin, or bradykinin (Zugaza et al., 1997). This makes GPR30 a good candidate for the triggering of PKD1 phosphorylation upon BPA stimulation. Moreover, it cannot be excluded that, due to its hydrophobic properties, BPA may also interact with the hydrophobic transmembrane domain of another receptor and modulate its activity as does DTT with the FSHR (Munier et al., 2016), or directly bind to and regulate intracellular proteins leading to the stimulation of PKD1 activators (Berna et al., 2007; Doppler and Storz, 2007). How BPA affects cell proliferation and, more widely, cell behavior through PKD1 phosphorylation remains an open question. As mentioned earlier, BPA was shown to regulate different signaling pathways such as ERK (Dong et al., 2011; Song et al., 2015), EGFR (Sauer et al., 2017), FAK, and Src (Castillo et al., 2016). BPA was also described to modulate the expression of both cell-cycle related genes (Lee et al., 2012) and miRNA (Tilghman et al., 2012) in MCF-7 cells. Similarly, PKD1 was shown to regulate numerous signaling pathways [reviewed in (Youssef and Ricort, 2019)] and to be regulated through miRNA (Kim do et al., 2016). Among all the BPA and PKD1 targets, ERK appears as a common element which could be more accurately analyzed in order to decipher whether it may act downstream of phosphorylated-PKD1 under BPA exposure.

Finally, the maximal response was obtained for different BPA concentrations, varying from 10^−7^ to 10^−10^ M. Efficient BPA concentrations that significantly stimulate cell proliferation appear a little bit lower than previously described by others in MCF-7 cells (Lee et al., 2012; Tilghman et al., 2012; Zhang et al., 2012). Such a difference can be attributed to the existence of MCF-7 sub-clones that express different combinatory of receptors and mediators of BPA signaling (knowing that this cell line tends to derive rapidly). Observed discrepancies may also reside in the experimental conditions used by authors. In fact, we observed that cell density (unpublished data) is a crucial point determining whether and how cells are responsive or not to BPA. This suggests that cell to cell interactions and/or secreted factors may also affect cell response to BPA. All these parameters may account for the differences in cell responses to BPA (and other EDs acting similarly) and explain non-monotonic responses reported by others (Lagarde et al., 2015). Optimal BPA concentrations may also vary depending on the biological effect analyzed (cell proliferation, anchorage-independent growth, PKD1 phosphorylation, etc.). This important point illustrates the involvement of multiple parameters, such as direct and indirect BPA targets and the complexity of the signaling pathways involved. In fact, it is not appropriate to make direct and trivial comparisons between short-term (e.g. rapid protein phosphorylation) and long-term effects (e.g. cell proliferation or tumor growth), because they involve different kinetics and regulatory mechanisms. Thus, the complexity of intracellular signaling pathways and the abundance of regulated molecular partners cannot be directly connected, since long-term effects require more profound and sustainable cellular changes than short-term effects. Thus, small and immediate responses, such as protein phosphorylation, may be the starting point of complex cellular responses, such as the induction of cell proliferation. This is important because the optimal concentration for BPA-induced PKD1 phosphorylation, ranging from 10^−11^ to 10^−10^ M, makes PKD1 one of the first targets of this compound and one of the first initiators of the response to BPA. In addition, the differences observed in the dose responses may also be due to the diversity of the molecular targets of BPA, each with their own specific affinity towards this compound. The complexity of the adverse effects induced by BPA, as a paradigm of endocrine disruptors, makes the study of its cellular and physiological effects challenging because the precise signaling pathways activated upon exposure to BPA are still mostly unknown, despite the characterization of its binding to various receptors.

Our data provide new insight into the molecular mechanisms regulated by BPA and the signaling pathways it activates. By identifying PKD1 as a functional non-genomic target of BPA both *in vivo* and *in vitro*, we provide an important step forward in understanding the molecular mechanisms involved in tumor development regulated by this endocrine disruptor.

## Ethics Statement

This study was carried out in accordance with the recommendations of institutional committees of the French Ministry of Agriculture (A-75-06-12). The protocol (75-06-12) and the project (#4028) were approved by the animal house of the Centre de Recherche des Cordeliers (namely Centre d’Exploration Fonctionnelle) where the animal studies were carried out.

## Author Contributions

MM-L led the project and performed experiments. CL, AB, IY, SLoi, and SLog performed experiments. SB and J-MR conceived and directed the project, designed the experiments, supervised the participants, and wrote the manuscript.

## Funding

This work was supported by the French National Center for Scientific Research (CNRS), the Ecole Normale Supérieure Paris-Saclay, the National Institute for Health and Medical Research (INSERM), the GEFLUC Paris-IdF (grant to J-MR), and the Fondation Santé Environnementale de la Mutuelle Familiale, Paris, France—AP-FSE-17-001 (grant to SB).

## Conflict of Interest

The authors declare that the research was conducted in the absence of any commercial or financial relationships that could be construed as a potential conflict of interest.
